# Sortase A-Cleavable CD1d Identifies Sphingomyelins as Major Class of CD1d-Associated Lipids

**DOI:** 10.3389/fimmu.2022.897873

**Published:** 2022-07-07

**Authors:** Maren Rudolph, Yuting Wang, Theresa Simolka, Emilie Huc-Claustre, Lingyun Dai, Gijsbert Grotenbreg, Gurdyal Singh Besra, Anna Shevchenko, Andrej Shevchenko, Sebastian Zeissig

**Affiliations:** ^1^ Department of Medicine I, University Medical Center Dresden, Technische Universität (TU) Dresden, Dresden, Germany; ^2^ Center for Regenerative Therapies Dresden (CRTD), Technische Universität (TU) Dresden, Dresden, Germany; ^3^ Max Planck Institute of Molecular Cell Biology and Genetics, Dresden, Germany; ^4^ Department of Geriatrics, First Affiliated Hospital of Southern University of Science and Technology (Shenzhen People’s Hospital), Shenzhen, China; ^5^ Maze Therapeutics, South San Francisco, CA, United States; ^6^ School of Biosciences, University of Birmingham, Birmingham, United Kingdom

**Keywords:** CD1d, NKT cell, Sortase A, shotgun lipidomics, sphingomyelin

## Abstract

CD1d is an atypical MHC class I molecule which binds endogenous and exogenous lipids and can activate natural killer T (NKT) cells through the presentation of lipid antigens. CD1d surveys different cellular compartments including the secretory and the endolysosomal pathway and broadly binds lipids through its two hydrophobic pockets. Purification of the transmembrane protein CD1d for the analysis of bound lipids is technically challenging as the use of detergents releases CD1d-bound lipids. To address these challenges, we have developed a novel approach based on Sortase A-dependent enzymatic release of CD1d at the cell surface of live mammalian cells, which allows for single step release and affinity tagging of CD1d for shotgun lipidomics. Using this system, we demonstrate that CD1d carrying the Sortase A recognition motif shows unimpaired subcellular trafficking through the secretory and endolysosomal pathway and is able to load lipids in these compartments and present them to NKT cells. Comprehensive shotgun lipidomics demonstrated that the spectrum and abundance of CD1d-associated lipids is not representative of the total cellular lipidome but rather characterized by preferential binding to long chain sphingolipids and glycerophospholipids. As such, sphingomyelin species recently identified as critical negative regulators of NKT cell activation, represented the vast majority of endogenous CD1d-associated lipids. Moreover, we observed that inhibition of endolysosomal trafficking of CD1d surprisingly did not affect the spectrum of CD1d-bound lipids, suggesting that the majority of endogenous CD1d-associated lipids load onto CD1d in the secretory rather than the endolysosomal pathway. In conclusion, we present a novel system for the analysis of CD1d-bound lipids in mammalian cells and provide new insight into the spectrum of CD1d-associated lipids, with important functional implications for NKT cell activation.

## Introduction

Cluster of differentiation 1 d (CD1d) is a major histocompatibility complex (MHC) class I-like molecule that binds and presents exogenous and endogenous lipids to a subset of T cells called natural killer T (NKT) cells. NKT cells rapidly and strongly respond to the recognition of cognate antigens among CD1d-associated lipids and play important roles in antimicrobial and cancer immunity as well as in the regulation of chronic immune-mediated and autoimmune diseases (1–5).

Heterodimers of CD1d and β2-microglobulin are assembled in the endoplasmic reticulum (ER) followed by trafficking of CD1d through the secretory pathway to the plasma membrane, where bound lipids are presented to NKT cells (6, 7). In addition, through a tyrosine-based sorting motif in its cytoplasmic tail, CD1d is recruited to the endolysosomal system, where bounds lipids are exchanged against other endogenous and exogenous lipids, followed by return of CD1d to the cell surface and presentation of associated lipids (7, 8). As such, CD1d broadly surveys different cellular compartments for self- and non-self-derived lipids. Lipid loading onto CD1d and the exchange of bound lipids is facilitated by lipid transfer and editing proteins such as the microsomal triglyceride transfer protein (MTP) and saposins (9–18). However, little is known about whether these lipid transfer and editing proteins actively shape the repertoire of lipids associated with CD1d or merely facilitate the loading of the most abundant lipids in the respective compartments.

Given the central role of CD1d in the activation of NKT cells, the identification of CD1d-associated lipids has received great interest. Initial work aiming to characterize the spectrum of CD1d-bound lipids used detergent-based extraction of CD1d (19). Subsequent studies used engineered secreted CD1d proteins which lacked the transmembrane and intracellular domains of CD1d and therefore allowed to harvest CD1d from the supernatant of transfected cells (19–24). This avoided the need for detergent-based extraction of CD1d and inadvertent loss of bound lipids associated with the use of detergents. These studies revealed that in accordance with its broad survey of cellular compartments and its ability to bind a wide spectrum of structurally different lipids, CD1d associates with various different glycerophospholipids and sphingolipids including diacylglycerols, plasmalogens, lysophospholipids and cardiolipins (21). While providing important insight into the repertoire of CD1d-associated lipids, the use of secreted CD1d proteins had two major limitations. First, secreted CD1d not only lacked the transmembrane domain of CD1d but also its cytoplasmic tail. Therefore, secreted CD1d did not traffic through the endolysosomal system and therefore did not allow to study lipids loaded onto CD1d in these compartments. Moreover, the use of secreted CD1d to identify associated lipids was restricted to cultured cells *in vitro* and did not allow to assess lipids bound to CD1d *in vivo* or *ex vivo*.

To address these limitations, recent studies engineered CD1d proteins to contain recognition sites of endogenous (23) or exogenous (22) proteases. These constructs were designed to maintain the transmembrane and intracellular domains of CD1d and to preserve CD1d trafficking, while enabling protease-based release of CD1d from intact cells for analysis of CD1d-bound lipids. Yuan et al. (23) used a human CD1d construct containing an interferon (IFN)-γ-inducible lysosomal thioreductase (GILT) site aiming for spontaneous GILT-based release of CD1d in the lysosome. While endogenous GILT-based cleavage was only observed for a minor fraction of engineered CD1d and did not release sufficient amounts of CD1d for mass spectrometry (MS)-based lipidomics, extraction of plasma membranes of transfected cells and subsequent papain treatment allowed to release CD1d for affinity purification and MS-based lipidomics of associated lipids. Papain-cleavable CD1d (pclCD1d) showed predominant association with cellular phosphatidylcholine (PC), the major cellular lipid of the CD1d-expressing cell line. In addition, low amounts of sphingomyelin (SM) and lysophospholipids were also found to be associated with CD1d. This work suggested that the spectrum of CD1d-associated lipids is largely reminiscent of the cellular lipid pool. Surprisingly, however, lipids associated with pclCD1d differed substantially from secreted CD1d (secCD1d) in that secCD1d contained predominantly SM species, while pclCD1d predominantly associated with PC species. The authors therefore suggested that pclCD1d-associated PCs were acquired in the endolysosomal rather than the secretory pathway. Studies by Muindi et al. (22) extended this work by analyzing glycosphingolipids (GSL) associated with murine CD1d containing a tobacco etch virus (TEV) protease site in the extracellular juxtamembrane region of CD1d. While these studies aimed for TEV protease-based release of CD1d, substantial spontaneous release of CD1d was observed in TEV buffer in the absence of active TEV protease. However, high performance liquid chromatography (HPLC)-based analysis of CD1d released either spontaneously or with the use of TEV protease revealed differences in the GSL repertoire associated with TEV- compared to secCD1d. Similar to findings of glycerophospholipids and sphingolipids by Yuan et al. (23), GSLs associated with TEV-CD1d were largely representative of the cellular GSL content, while the spectrum of GSLs associated with secCD1d differed from that of the cellular GSL content.

Together, these studies suggested that glycerophospholipids and GSLs associated with CD1d proteins that retain the subcellular trafficking patterns of endogenous CD1d are largely reminiscent of the cellular lipid pool. However, a detailed, comprehensive, and quantitative assessment of CD1d-associated lipids was not provided. In addition, due to the technical issues associated with pclCD1d and TEV-CD1d constructs, neither of the approaches allowed for protease-based harvesting of CD1d from intact cells in the absence of spontaneous CD1d release. Here, we therefore developed CD1d proteins containing the recognition motif of *Staphylococcus aureus* Sortase A (SortA), a transpeptidase which allows for simultaneous cleavage and covalent linkage of affinity tags to target proteins (sortagging) (25, 26). We show that SortA-cleavable CD1d shows unimpaired cellular trafficking and antigen presentation and enables proteolytic cleavage of CD1d from intact, living cells. Using this system, we assessed CD1d-associated lipids by quantitative shotgun lipidomics. We demonstrate that under constitutive conditions, CD1d is associated with a limited number of sphingolipids and glycerophospholipids, which are loaded onto CD1d in the secretory pathway. Lipids associated with CD1d are biased towards long-chain sphingo- and glycerophospholipids which stably associate with CD1d but represent minor lipid species in total cellular lipidomes.

## Materials and Methods

### Sortase A-Cleavable mCD1d and mH-2Kb Constructs

Sortase A-cleavable constructs were generated by inserting the SortA peptide recognition motif LPETG together with a glycine-/serine-based linker between the extracellular and the transmembrane domain of mouse *Cd1d1* or *H-2Kb*. The linker sequence was required to allow for access of SortA and no SortA-dependent cleavage of CD1d was observed in the absence of this linker sequence. To investigate how endolysosomal trafficking affects the spectrum of CD1d-associated lipids, we also generated a SortA-cleavable CD1d construct lacking the cytoplasmic tail required for endolysosomal trafficking of CD1d (tail-deleted (TD)-SortA-CD1d). To this end, a stop codon was inserted directly 3’ of the transmembrane domain of CD1d. The sequences were cloned into the pcDNA3.1(+) expression vector. An empty pcDNA3.1(+) vector served as negative control (mock).

### Cell Lines Stably Expressing SortA-CD1d and SortA-H-2Kb

L cells, a murine fibroblast cell line, was obtained from American Type Culture Collection (ATCC). SortA-CD1d/H-2Kb constructs were transfected using LipofectamineTM 2000 (Thermo Fisher Scientific), and stable clones were isolated by limiting dilution under selection with G418 (Carl Roth). Protein cell surface expression of stable clones was analyzed by flow cytometry using phycoerythrin anti-mouse CD1d and phycoerythrin anti-H-2Kb antibodies (Biolegend).

### Immunofluorescence Staining and Confocal Laser Scanning Microscopy

Cells were grown on coverslips, washed in ice cold phosphate buffered saline (PBS), fixed in 4% paraformaldehyde in PBS, washed and permeabilized in PBS containing 0.02% saponin and 0.2% glycine. For blocking, PBS containing 10% fetal bovine serum (FBS) and 0.02% saponin was used before the first antibody was applied for 12 h at 4°C in blocking solution. Cells were washed in PBS/0.02% saponin and the secondary antibody and DAPI were added for 1 h at room temperature (RT). After washing, FluorSave™ reagent (MerkMillipore) was mounted. Confocal microscopy was performed using a Zeiss confocal laser scanning microscope LSM 980/MP, equipped with an Airysacn 2 module and a Plan-APOCHROMAT 63×/1.4 Oil DIC immersion objective, of the Light Microscopy Facility, a Core Facility of the CMCB Technology Platform at TU Dresden.

### Antigen Presentation and Cytokine Quantification

KRN7000 (alpha-galactosyl ceramide, α-GalCer) was purchased from Avanti Polar Lipids, Inc. Gal(α1–2)galactosylceramide (GalGalCer) and C20:2 (α-GalCer analogue with 20 carbon di-unsaturated N-acyl chain) were prepared from d-lyxose, as described (27). All lipids were diluted at a concentration of 1 mg/ml in dimethyl sulfoxide (DMSO). L cells were seeded in 96-well plates at a density of 4 × 10^4^ cells per well and lipids were added after 6 h. Cells were washed three times with 1× RPMI-1640 medium (Thermo Fisher Scientific) and 1x10^5^ NKT hybridoma DN32.D3 (28) cells per well added. After 24 h, the supernatant was obtained and IL-2 concentrations determined using a mouse interleukin (mIL2) enzyme-linked immunosorbent assay (ELISA) (BD Bioscience) according to the manufacturer’s instruction.

### Cell Culture and Sortagging

Cell culture of L cells was performed in Corning^®^ CellBIND^®^ Surface HYPERFlask^®^ cell culture vessels according to the manufacturer’s recommendations. L cells were grown in DMEM (high glucose) (Thermo Fisher Scientific) supplemented with 10% FBS (Thermo Fisher Scientific) and 2 mM L-glutamine (Thermo Fisher Scientific). In order to harvest the cells, cells were washed with 1 × PBS and trypsinized. The cell suspension was centrifuged (300 × g, 5 min, RT), pelleted cells were resuspended in 1× PBS and counted. Cells were then washed twice using 10 ml of tris (hydroxymethyl) aminomethane (Tris) buffer (50 mM Tris-HCl, 150 mM NaCl, 1.8 mM CaCl_2_, pH 7.5 at 37°C). His-tagged Sortase (Δ59SortA) (29) was expressed using the pET28(a)-SortA vector and purified using HisTrap HP columns (GE Healthcare). The final SortA concentration was 25 µM. 3G-Twin-Strep-tag (GGGWSHPQFEKGGGSGGGSGGSAWSHPQFEK, purity ≥ 95%) (peptides&elephants) was added in a molar ratio of 1:25 of SortA to tag. The calcium concentration was adjusted to 1.8 mM. The cells were incubated with SortA for 3 h at 37 °C while shaking at 600 rpm. After incubation, the cells were centrifuged (300 × g, 5 min, 4°C). The collected cells were kept for flow cytometric analysis, while the supernatant containing cleaved mCD1d was further purified.

### Purification Twin-Strep-Tagged Proteins

The collected supernatant was centrifuged at 4,500 × g for 20 min at 4°C, and again centrifuged at 118,244 × g for 75 min at 4°C. SortA activity in the cell supernatant was inhibited by adding [2-(Trimethylammonium)ethyl] methanethiosulfonate chloride (MTSET) (Toronto Research Chemicals) (final conc. 5 mM). Samples were dialyzed against Tris buffer (100 mM Tris-HCl pH 8 at RT, 150 mM NaCl, 1 mM EDTA) and concentrated. Purification of tagged proteins was done using Strep-Tactin^®^ Superflow high capacity^®^ 50% suspension (Iba) as resin material. The purification was performed according to manufacturer’s instructions.

### ELISA-Based Quantification of CD1d and β2-Microglobulin

For quantification of β2-microglobulin (β2M), a direct ELISA using anti-β2M antibody (AA1-119) (antibodies-online.com) was performed. 96 well plates (Corning^®^ Costar^®^ 96 well EIA/RIA plate, flat bottom) were coated with standard mCD1d monomer (NIH Tetramer Core Facility) and diluted samples in coating buffer (0.1 M sodium carbonate, pH 9.5) 14 h at 4°C. For the standard curve, a serial dilution of mCD1d-β2M heterodimers (40 ng/mL) (NIH Tetramer Core Facility) was used. Wells were washed trice with washing buffer (PBS, 0.05% Tween 20) and blocked for 2 h at RT with blocking buffer (PBS, 1% FBS). Wells were washed trice with washing buffer and anti-β2M antibody (AA1-119) (antibodies-online.com) (1:500) was added in blocking buffer for 2 h at RT. Wells were washed trice with washing buffer and anti-rabbit HRP conjugate (Cell Signaling Technology) (1:2500) was added in blocking buffer for 80 min at RT. Wells were washed five times with washing buffer. 3, 3’, 5, 5’ - Tetramethylbenzidine (TMB) reagent (BioLegend) was added and the reaction stopped with 1M HCl. Plates were read at 450 nm using a FlexStation 3 multi-mode microplate reader (Molecular Devices). After background subtraction, β2m concentrations were determined for mCD1d and H-2Kb samples.

### Protein Identification by LC-MS/MS

Purified proteins were separated by 12.5% SDS PAGE and stained using Coomassie blue. Visible bands were excised, cut into 1 × 1 mm cubes and incubated consequently with 10 mM dithiothreitol and 55 mM iodacetamide in 100 mM ammonium bicarbonate. In-gel digestion was performed overnight using trypsin (12 ng/µl, Promega) at 37°C. The resulting peptide mixtures were extracted twice by exchange using 5% formic acid (FA) and acetonitrile, extracts pooled together and dried in a vacuum centrifuge. Peptides were re-suspended in 25 µl of 5% FA and a 5 µl aliquot was analyzed by LC-MS/MS on a nano-ultra performance liquid chromatography (UPLC) system interfaced to a Q Exactive HF Orbitrap mass spectrometer (both Thermo Fisher Scientific). The nano-UPLC was equipped with an Acclaim PepMap100 C18 75 μm i.d. x 20 mm trap column and 75 μm x 15 cm analytical column (3 μm/100A, Thermo Fisher Scientific). Peptides were separated using 80 min linear gradient: Solvent A was 0.1% aqueous formic acid and Solvent B was 0.1% FA in neat acetonitrile. Three blank runs were performed after each sample analysis to minimize carryover. Spectra were acquired by data-dependent acquisition method using Top20 approach and dynamic exclusion time of 15 s. Lock mass was set to the singly charged ion of dodecamethylcyclohexasiloxane ion ((Si(CH3)2O))6; *m/z* = 445.1200). The acquired spectra were searched using Mascot software (v. 2.2.04) against UniProt database (April 2018) and the results evaluated by Scaffold software v.4.7.5 (Proteome Software, Portland). Protein hits were accepted if matched with two peptides and proteins and spectra False Discovery Rates (FDR, function of the Scaffold software) was below 1%.

### Lipid Extraction

Synthetic lipid standards were purchased from Avanti Polar Lipids, Inc. Internal standard mix was prepared in methyl-*tert*-butyl ether (MTBE) or MTBE/MeOH (5:1.5; v/v), each containing cholesterol ester (CE)-D7 16:0, cholesterol (Chol)-D7, triglyceride (TG)-D5 50:0, diglyceride (DG)-D5 34:0, phosphatidylcholine (PC) 25:0, phosphatidylethanolamine (PE) 25:0, phosphatidylinositol (PI) 25:0, phosphatidylglycerol (PG), phosphatidic acid (PA) 25:0, lyso-phosphatidylcholine (LPC) 13:0, lyso-phosphatidylethanolamine (LPE) 13:0, lyso-phosphatidylinositol (LPI) 13:0, lyso-phosphatidic acid (LPA) 13:0, ceramide (Cer) 30:1:2, sphingomyelin (SM) 30:1:2 and galactosylceramide (GalCer) 30:1:2, lactosylceramide 30:1:2, and stored at −20°C until the analysis. The annotation of lipid species was performed according to (30). Glycerolipid and glycerophospholipid species were annotated by the number of carbon atoms:double bonds in all fatty acid moieties. Sphingolipid species were annotated by the number of carbon atoms:double bonds:hydroxyl groups at the ceramide backbone.

Lipid extraction was adapted from (31). 500 μl internal standard mix in MTBE was added to an equal volume of elution fractions of purified CD1d and H-2Kb. Blank elution buffer was used as background control. To extract lipid from cell lines, 700 μl of a mixture of internal standards in MTBE/MeOH (5:1.5; v/v) was added to the cells containing an equivalent of 50 μg of total protein. The upper organic phases were vacuum-dried and re-suspended in isopropanol/methanol/chloroform 4:2:1 (v/v/v) containing 7.5 mM ammonium formate and proceeded to mass spectrometric analyses.

### Lipid Identification and Quantification by Shotgun Lipidomics

Mass spectrometric analyses were performed as previously described (32). Briefly, analysis was performed on a Q Exactive instrument (Thermo Fisher Scientific) equipped with a robotic nanoflow ion source TriVersa NanoMate (Advion BioSciences) using nanoelectrospray chips with the diameter of spraying nozzles of 4.1 μm. The ion source was controlled by the Chipsoft 8.3.1 software (Advion BioSciences). Ionization voltage was +0.96 kV in positive and −0.96 kV in negative mode; backpressure was 1.25 psi in both modes by polarity switching. The temperature of the ion transfer capillary was set to 200°C; S-lens RF level was 50%. For targeted-selected ion monitoring (t-SIM), spectra were acquired within the range of *m/z* 350–1000 in positive and negative ion mode, respectively, at the target mass resolution of R*_m/z 200_* = 140,000 and with automated gain control (AGC) of 5 × 10^4^, maximum injection time of 650 ms, and isolation window of 20 Th. Free cholesterol (Chol) was quantified by parallel reaction monitoring (PRM) Fourier transform (FT) MS/MS in positive mode. For FT MS/MS, the number of microscans was set to 1, isolation window to 0.8 Da, normalized collision energy to 12.5%, AGC to 5 × 10^4^ and maximum injection time to 3s. Spectra were subjected to repetition rate filtering and t-SIM spectra were stitched together using Peakstrainer (33). Lipids were identified using LipidXplorer (34)Molecular Fragmentation Query Language (MFQL) queries were compiled for identifying PC, ether phosphatidylcholine (PC O-), LPC, ether lyso-phosphatidylcholine (LPC O-), PE, ether phosphatidylethanolamine (PE O-), LPE, PI, LPI, PG, PA, LPA, Cer, SM, hexosylceramide (HexCer), di-hexosylceramide (DHCer), Chol, CE, TG, and DG lipid classes. Lipids were identified by their accurately determined intact m/z (mass accuracy better than 5 ppm) and quantified by comparing the isotopically corrected abundances of their molecular ions with the abundances of internal standards of the same lipid class. Only lipids whose monoisotopic peaks were detected with a signal-to-noise ratio to background control above the value of 5 were quantified. Lipid amount in elution fractions was normalized to protein amount and reported as pmol per pmol of purified H-2Kb or CD1d. Lipid amount in cell lines was normalized to total lipids and reported as molar fraction. MS^2^ validation was applied to identified CD1d-bound lipids. The acquisition of PRM FT MS/MS spectra was adapted from Schuhmann et al. (35) SMs were identified by the phosphocholine head group fragment *m/z* 184.074 from the [M+H]^+^ precursors and demethylation fragments [M-CH_3_]^-^ as neutral loss of Δ*m/z* = - 60.021 from the [M+ HCOO]^-^ precursors. PCs were identified by the phosphocholine head group fragment *m/z* 184.074 from the [M+H]^+^ precursors and fatty acyl fragments from the [M+ HCOO]^-^ precursors. Cer and HexCer were identified by the sphingoid backbone and fatty acyls.

### Statistical Analysis

Lipids associated with CD1d or H-2Kb were assessed in four independent experiments of sortagging, purification and shotgun lipidomics. Lipids were considered specifically associated with to SortA-CD1d or SortA-TD-CD1d when they fulfilled the following criteria: 1. Lipids must be detected in purifications of SortA-CD1d or SortA-TD-CD1d in at least three out of four independent experiments. 2. The mean value of lipid abundance must be at least three-fold higher in SortA-CD1d or SortA-TD-CD1d purifications compared to SortA-H-2Kb control. The Kruskal-Wallis test together with the Dunn’s test was used to determine the significance of changes in lipid abundance among the elution fraction of SortA-H-2Kb, SortA-CD1d and SortA-TD-CD1d, as well as among the three types of cell lines. Correction of the P values for multiple comparisons was done by the Benjamini-Hochberg approach. All calculations were performed using R4.1.2. For bar graphs, error bars indicate the standard error of the mean (SEM). * equals p<0.05; ** equals p< 0.01.

## Results

### SortA-Cleavable-CD1d Shows Unimpaired Trafficking and Antigen Presentation

*S. aureus* SortA is a transpeptidase which recognizes a five amino acid-based motif (LPETG). Following recognition of its binding motif, SortA forms an acyl-enzyme intermediate and subsequently links the cleaved protein covalently to a N-terminal triglycine motif (25, 36). As such, sortagging allows for combined cleavage of target proteins and covalent linkage of a triglycine-containing affinity tag. Importantly, SortA exerts its functions under physiologic pH conditions in cell culture media and Tris-buffered solutions thus allowing for proteolytic processing and tagging of cell surface proteins using live mammalian cells (36–39).

**Figure 1:**
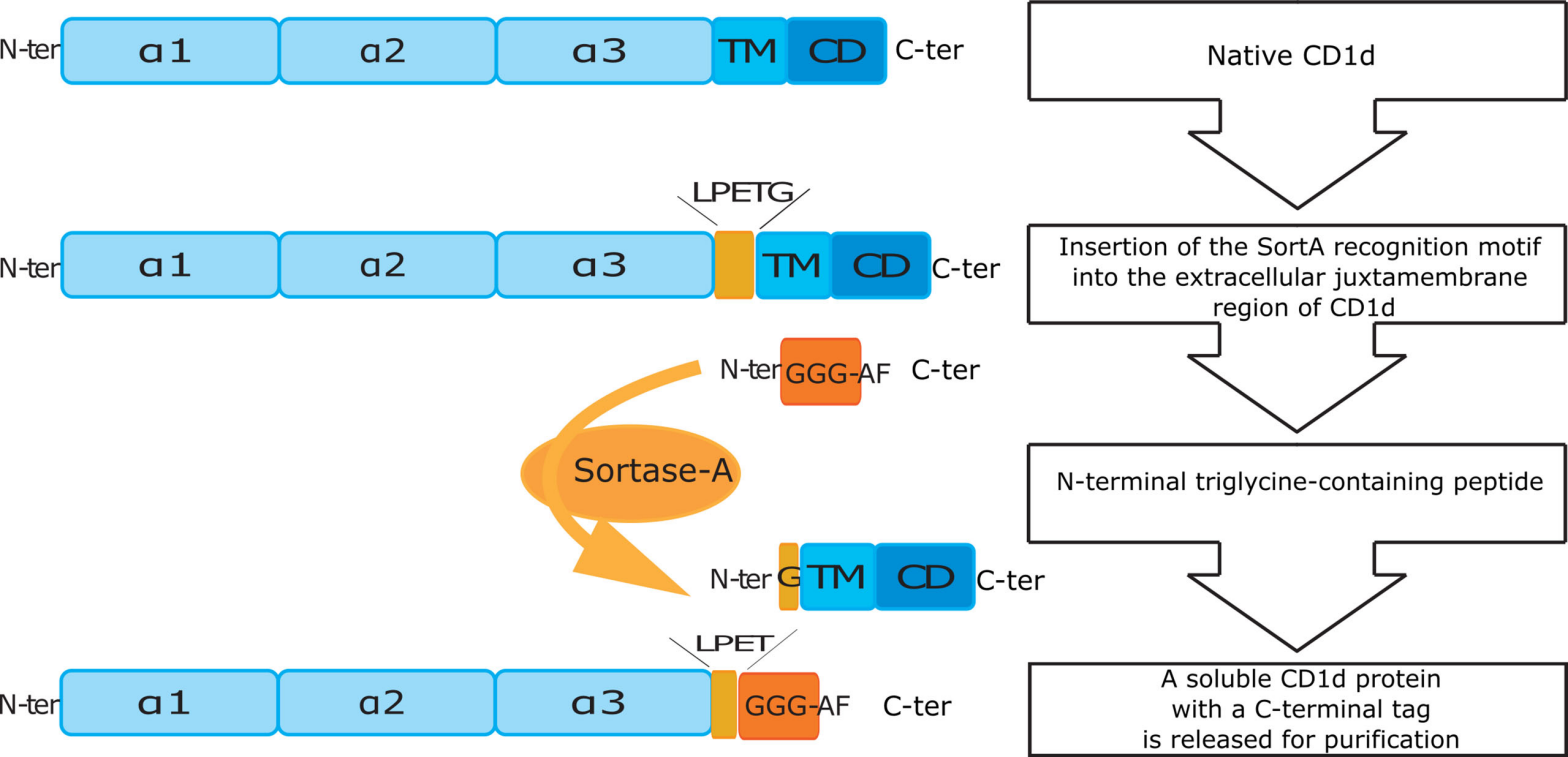
Schematic overview of cloning and purification of SortA-CD1d. N-ter/C-ter: N-terminal and C-terminal end of peptides and proteins; α1-3: alpha helical domain 1-3 of CD1d; TM, transmembrane domain; CD, cytosolic domain; LPETG, five amino acid long recognition motif of Sortase A; GGG-AF, triglycine labeled affinity tag.

To investigate the spectrum of CD1d-associated lipids, we engineered CD1d proteins containing the SortA recognition motif in the extracellular, juxtamembrane region of mouse CD1d (**Figure 1**). In order to gain insight into the cellular compartments in which specific lipids are loaded onto CD1d, we also generated SortA-CD1d proteins which lacked the tyrosine-based sorting signal (YQDI) required for recruitment of CD1d into the endolysosomal pathway (40, 41). These tail-deleted (TD)- CD1d proteins allowed to selectively study lipids loaded onto CD1d in the secretory pathway and/or at the cell surface. CD1d and classical MHC class I molecules share a common structure of heterodimeric transmembrane proteins associated with β2-microglobulin, which traffic through the secretory pathway to the cell surface (42). Since MHC class I binds peptides, while CD1d associates with lipids, MHC class I proteins are suitable controls to assess whether identified lipids are indeed specifically bound to CD1d or result from experimental contaminations by cellular lipids during the purification procedure. We therefore also generated engineered mouse MHC class I (SortA-H-2Kb) as control.

**Figure 2:**
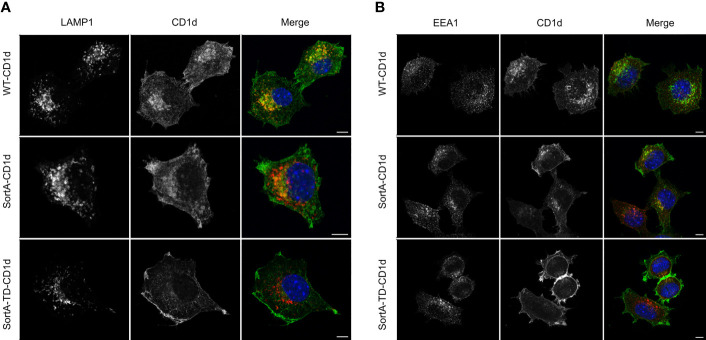
SortA-CD1d but not SortA-TD-CD1d traffics through the endolysosomal system. L cells stably transfected with WT-CD1d, SortA-CD1d or SortA-TD-CD1d were stained with antibodies against the lysosomal marker LAMP1 (**A**, red) or the early endosomal marker EEA1 (red, **B**). Cells were stained with antibodies against CD1d(green, **A, B**) and nuclei were counterstained with DAPI (blue). Scale bars represent 5 μm. Figures are representative of 2 independent experiments.

These constructs were used to generate mouse cell lines stably expressing SortA-CD1d, SortA-TD-CD1d and SortA-H-2Kb. We first investigated the subcellular distribution of SortA-CD1d and SortA-TD-CD1d. SortA-CD1d showed a subcellular distribution reminiscent of wildtype mCD1d (mouse *Cd1d1*), with localization both at the plasma membrane as well as colocalization with markers of early endosomes (EEA1) and lysosomes (LAMP1) (**Figures 2A, B**). In contrast, SortA-TD-CD1d was largely excluded from endosomal and lysosomal compartments with predominant localization at the plasma membrane as expected (**Figures 2A, B**).

**Figure 3:**
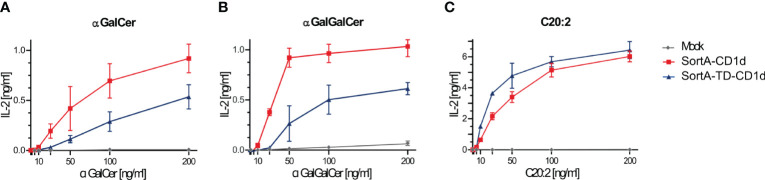
SortA-CD1d and SortA-TD-CD1d can load lipid antigens and present them to NKT cells. L cells stably transfected with SortA-CD1d or SortA-TD-CD1d were loaded with **(A)** αGalCer, **(B)** αGalGalCer or **(C)** C20:2 and were subsequently co-cultured with NKT hybridoma cells. IL-2 concentration as a measure of NKT cell activation was determined by ELISA in cell culture supernatants. Graphs show mean ± SD of biological duplicates and are representative for one out of at least two independent experiments.

We further tested the ability of SortA-CD1d and SortA-TD-CD1d to present lipid antigens to NKT cells. To this end, we investigated the presentation of α-galactosyl ceramide (α-GalCer), galactose (α1-2) α-galactosyl ceramide (α-GalGalCer) and C20:2 (α-GalCer analogue with 20 carbon di-unsaturated N-acyl chain), each having a distinct loading preference. C20:2 preferentially loads on the cell surface and does not require endolysosomal trafficking of CD1d for loading and presentation (27). α-GalCer can be loaded onto CD1d at the cell surface, but its loading is greatly facilitated in the endolysosomal environment (43). Lastly, α-GalGalCer is strictly dependent on endolysosomal processing before it can be loaded as α-GalCer onto CD1d (44). Lipids were added to cell lines stably expressing SortA-CD1d and SortA-TD-CD1d and antigen presentation was evaluated by measurement of IL-2 release by co-cultured NKT cell hybridomas. SortA-CD1d showed dose-dependent activation of all three lipids to NKT cells (**Figure 3**). In contrast, SortA-TD-CD1d showed substantially impaired NKT cell activation in response to loading with α-GalCer and α-GalGalCer, while the presentation of C20:2 did not differ between SortA-CD1d and SortA-TD-CD1d (**Figure 3**). As such, SortA-CD1d and SortA-TD-CD1d are functional CD1d proteins able to load and present lipid antigens to NKT cells. While SortA-CD1d can traffic through endolysosomal compartments and load lipids in these compartments, TD-CD1d is largely excluded from the endolysosomal pathway.

### Sortagging Allows to Harvest and Label CD1d and MHC Class I From Live Mammalian Cells

**Figure 4:**
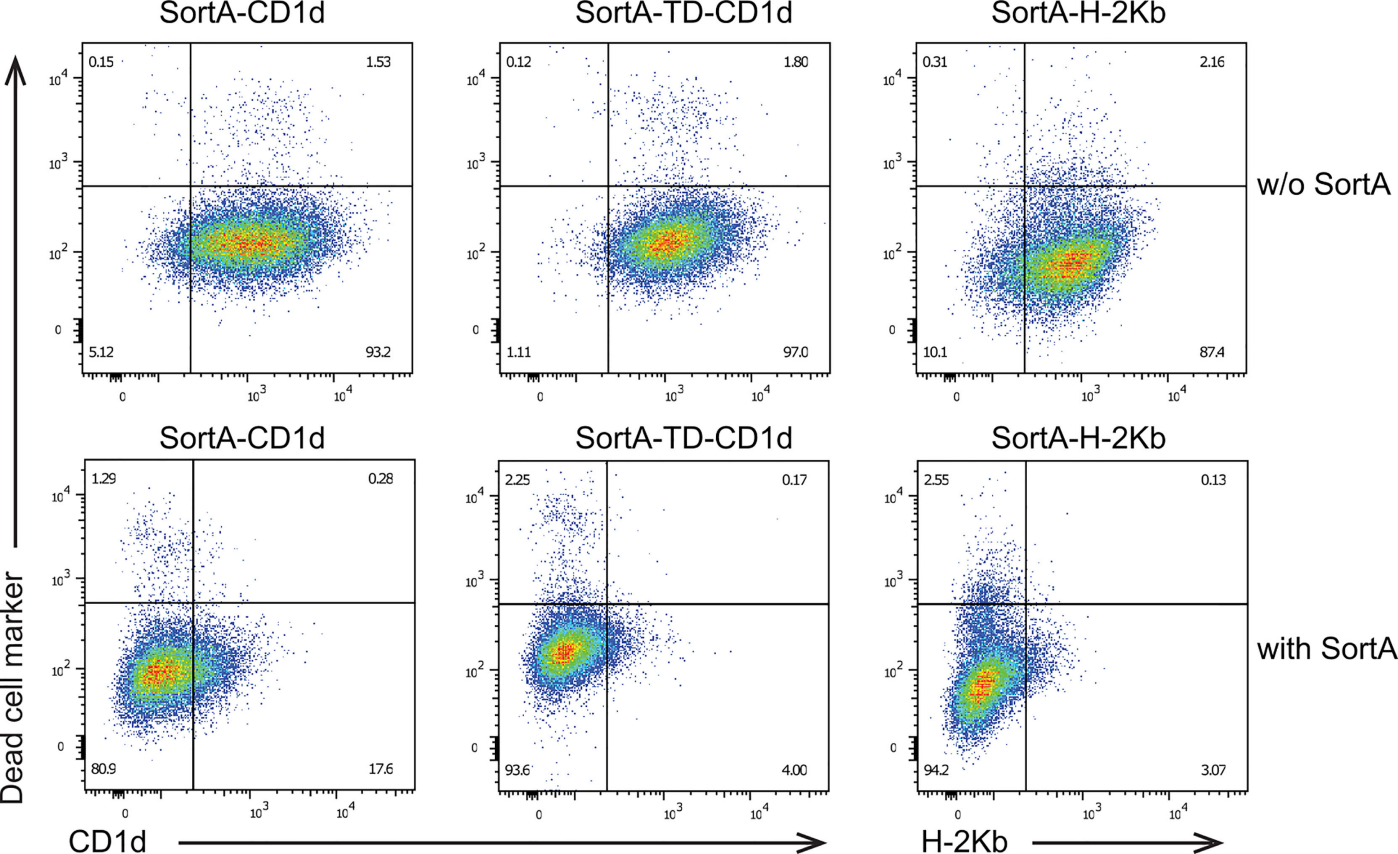
Sortase A efficiently cleaves cell surface CD1d and H-2Kb without affecting cell viability. L cells stably transfected with SortA-CD1d, SortA-TD-CD1d, and SortA-H-2Kb were incubated in the presence or absence of SortA. Representative pseudocolor plots of cells stained with phycoerythrin-labeled antibodies against CD1d or H-2Kb and Zombie VioletTM as dead live marker are shown. w/o, without. Results are representative of 4 independent experiments.

We next examined whether SortA can cleave cell surface CD1d and H-2Kb and whether exposure to SortA affects cell viability. To this end, we performed flow cytometry-based analysis of cells incubated with and without SortA and a triglycine-containing Twin-Strep-tag (**Figure 4**). At baseline, SortA-CD1d, SortA-TD-CD1d, and SortA-H-2Kb were abundantly expressed by stably transfected L cells. This was in line with results of immunofluorescence and antigen presentation studies and suggested negligible spontaneous cleavage and loss of proteins containing the SortA recognition motif. Exposure to SortA led to efficient proteolytic cleavage of CD1d and H-2Kb from the cell surface, with the vast majority of cell surface CD1d and H-2Kb released after sortagging (**Figure 4**). Moreover, exposure to SortA did not affect cell viability (**Figure 4**).

**Figure 5:**
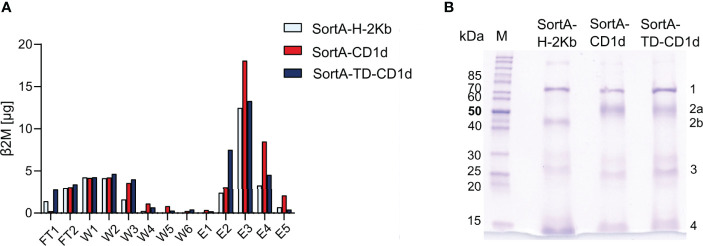
Sortase A allows for efficient cleavage, tagging, and purification of H-2Kb, CD1d, and CD1d-TD. **(A)** ELISA-based analysis of β2-microglobulin in different purification fractions. FT: flow through, W1-6: wash fractions, E1-5: elution fractions. **(B)** Coomassie blue-stained SDS gel of elution fraction 3 (E3) of SortA-H-2Kb, SortA-CD1d, and SortA-TD-CD1d. 1: mouse heat shock protein 70, 2a: mCD1d, 2b: H-2Kb 3: Sortase A, 4: β2m and Strep-Tactin^®^. M: PageRuler™ unstained protein ladder. Results are representative of 4 independent experiments.

To examine the efficiency of labeling of cleaved CD1d and H-2Kb, we purified supernatants of cells after sortagging using Strep-Tactin columns. To enable comparative quantification of CD1d and H-2Kb, we capitalized on the fact that both proteins form heterodimers with β2-microglobulin and assessed the β2-microglobulin concentration in flow-through, wash, and elution fractions (**Figure 5A**). These studies showed that β2-microglobulin-containing proteins were predominantly contained in elution fractions, while unlabeled β2-microglobulin-containing proteins contained in flow-through and wash fractions represented a minor fraction of total β2-microglobulin (**Figure 5A**). As such, these studies documented a high efficiency of Twin-Strep-tag-labeling.

To assess the purity of eluted proteins, we subjected eluted content to SDS-PAGE and protein mass spectrometry. Coomassie gels revealed bands representing mouse CD1d, H-2Kb and β2M, StrepTactin, SortA and mouse heat shock 70 kDa protein (HSP700) as identified by LC-MS/MS (**Figure 5B**). Recovery of mouse HSP70 was in line with previous work demonstrating that endogenous biotinylated proteins such as HSP70 are co-purified in Twin-Strep-II-based extractions (45–47). HSP70 was contained in similar amounts in H-2Kb, CD1d, and TD-CD1d purifications and potential confounding HSP70-associated lipids would therefore introduce a systematic error that can be accounted for by subtraction of H-2Kb-derived signals. Together, these results demonstrate that SortA-CD1d, SortA-TD-CD1d, and SortA-H-2Kb allow for efficient, simultaneous proteolytic cleavage and tagging of CD1d and H-2Kb from live mammalian cells for subsequent purification and mass spectrometry

### CD1d Preferentially Associates With Long-Chain Sphingolipids

**Figure 6:**
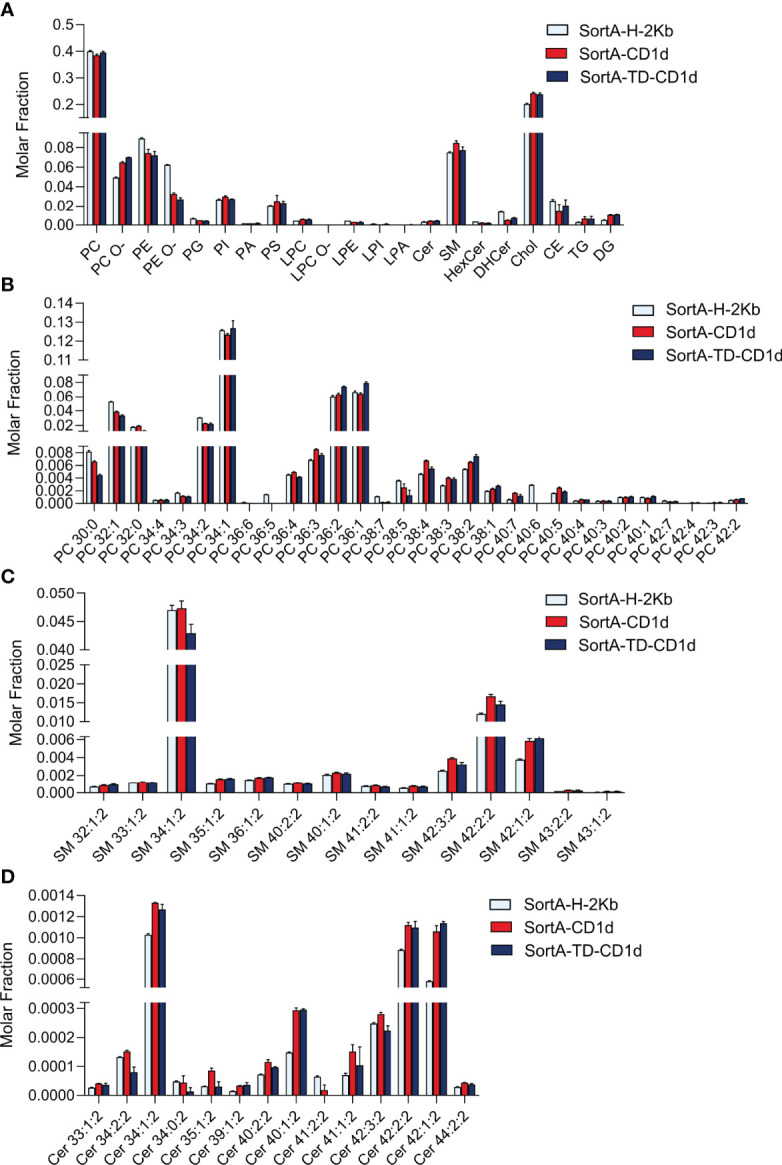
Comparative total cellular lipid profiles of L cells expressing CD1d, CD1d-TD or H2Kb. Total cellular lipids derived from L cells stably transfected with SortA-CD1d, SortA-TD-CD1d and SortA-H-2Kb were analyzed using shotgun lipidomics. Lipid classes **(A)** and lipid species from **(B)** PC, **(C)** SM and **(D)** Cer) are expressed as molar fraction (pmol lipid per pmol total lipids) with error bars indicating mean ± SEM. Statistics: Kruskal-Wallis, multi-test correction of p values was performed using the Benjamini-Hochberg correction. No significant differences in lipid abundance between cells expressing Sort-H-2Kb, SortA-CD1d and SortA-TD-CD1d were observed.

We next performed shotgun lipidomics of purified SortA-CD1d, SortA-TD-CD1d, and SortA-H-2Kb. Since H-2Kb binds peptides rather than lipids it served as a negative control to assess whether lipids are specifically bound to CD1d or released from the cells during the extraction and purification procedure. Four independent experiments of sortagging, purification, and shotgun lipidomics were performed. Lipids were considered specifically associated with SortA-CD1d and/or Sort-A-TD-CD1d when they fulfilled the following pre-specified criteria: 1. Lipids must be detected in purifications of SortA-CD1d or SortA-TD-CD1d in at least 3 of 4 independent experiments. 2. Lipids must show at least show three-fold higher abundance in SortA-CD1d or SortA-TD-CD1d purifications compared to SortA-H-2Kb. We first investigated whether total cellular lipidomes were comparable between cells expressing SortA-CD1d, SortA-TD-CD1d, and SortA-H-2Kb, an important requirement for comparative lipidomics of SortA-CD1d- and SortA H-2Kb-associated lipids. Shotgun lipidomics covered 291 lipid species from 21 lipid classes and indeed demonstrated comparable lipid compositions of the three cell lines with PC, Chol, and SM representing the three most abundant lipid classes (**Figure 6**).

**Figure 7:**
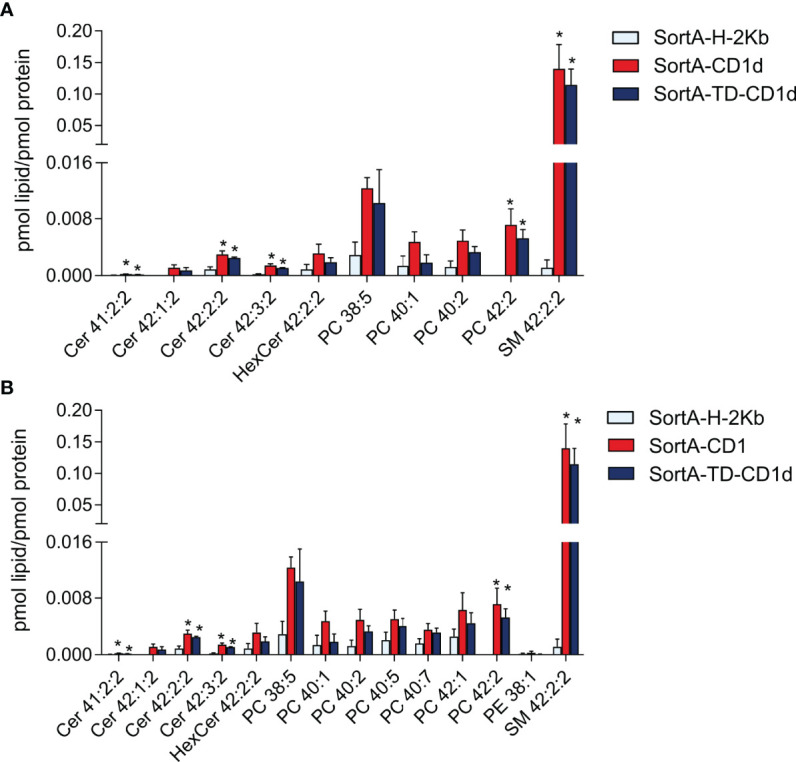
Lipids associated with SortA-CD1d and SortA-CD1d-TD. SortA-CD1d, SortA-TD-CD1d, and SortA-H-2Kb were purified as described in Methods and subjected to shotgun lipidomics. Only lipids identified in 3 of 4 independent experiments and showing **(A)** at least three -fold higher or **(B)** at least two-fold higher abundance in SortA-CD1d- or SortA-TD-CD1d purifications compared to SortA-H-2Kb purifications are shown. Lipid abundance is expressed as pmol lipid per pmol of the respective protein with error bars indicating mean ± SEM. Statistics: Kruskal-Wallis followed by Dunn´s *post-hoc* test, p-value * < 0.05; Asterisks indicate significance in the Dunn´s *post-hoc* test of a comparison of the group indicated by asterisk (SortA-CD1d or SortA-TD-CD1d) compared to SortA-H-2Kb.

Next, we performed shotgun lipidomics of purified SortA-CD1d, SortA-TD-CD1d, and SortA-H-2Kb with quantification of lipid abundance (expressed as pmol lipids/pmol CD1d/H-2Kb). Previous investigations of CD1d had reported vastly different numbers of CD1d-associated lipids ranging from a few dominant cellular lipids (19, 20, 22, 23) to several hundred lipid species (21, 24). With criteria defined as above, only 10 of 291 investigated lipids showed a specific association with SortA-CD1d compared to SortA-H-2Kb (**Figure 7A**). Lipids associated with SortA-CD1d included, in decreasing abundance, SM, PCs, ceramides, and HexCer. The major lipid species associated with SortA-CD1d was SM 42:2:2, whose abundance in CD1d purifications was more than 100-fold higher compared to the second most abundant CD1d-associated lipid, PC 38:5. Interestingly, with the potential exception of HexCer, all CD1d-associated lipids identified represent lipids not involved in the activation of NKT cells and therefore representing regulatory elements. Moreover, we did not observe any correlation between the abundance of lipids associated with SortA-CD1d compared to cellular lipid abundance. Indeed, the vast majority of SortA-CD1d-associated lipids represented lipids which are of low abundance in total cellular lipidomes (compare **Figures 6**, **7**). Investigation of SortA-CD1d-associated lipids further revealed predominant association of CD1d with lipids of long carbon chain length, a finding consistent with the observation that carbon chain length indirectly correlates with off-rates of lipids associated with CD1d (48).

Importantly, changing the cut-off values of the pre-specified filtering criteria did not have a major impact on the number or classes of lipids associated with CD1d. As such, reducing the threshold to lipids at least twice as abundant in SortA-CD1d- compared to SortA-H-2Kb purifications identified only four additional glycerophospholipid species specifically associated with SortA-CD1d, which again represented non-antigenic long-chain glycerophospholipids of low abundance in total cellular lipidomes (**Figures 6**, **7B**). In the absence of any filtering, numerous additional lipids were identified (**Supplementary Table 1**). However, these lipids showed largely similar abundance in H-2Kb purifications (**Supplementary Table 1**), suggesting that they were derived from membrane contaminations and did not reflect lipids truly bound to the two hydrophobic pockets of CD1d. As such, these findings highlight the value of using H-2Kb as a control and applying stringent filtering criteria to identify lipids specifically associated with CD1d. Together, our results demonstrate that under constitutive conditions, CD1d is associated with a limited number of lipids not representative of the total cellular lipidome and predominantly containing long-chain, non-NKT cell activating sphingolipids and PC.

### Disruption of Endolysosomal Trafficking Does Not Affect CD1d-Associated Lipids Under Constitutive Conditions

Past work has revealed substantial differences in the repertoire of lipids associated with secCD1d compared to pclCD1d or TEV-CD1d (22, 23). Specifically, secCD1d predominantly contained SM species, while pclCD1d with an intact endolysosomal pathway was predominantly associated with PCs (23). As such, it was suggested that SMs are loaded onto CD1d in the secretory pathway and subsequently replaced by PCs upon endolysosomal recycling (23). However, secCD1d and pclCD1d underwent different purification procedures, which may have affected the spectrum of CD1d-associated lipids (23). Our approach enabled us to directly compare the repertoire and abundance of lipids associated with SortA-CD1d compared to SortA-TD-CD1d, the latter of which is largely excluded from the endolysosomal pathway and predominantly surveys the secretory pathway. Intriguingly, different from previously work (23), we observed no differences in the spectrum and abundance of lipids associated with SortA-CD1d compared to SortA-TD-CD1d. Specifically, SM 42:2:2 was by far the most abundant lipid associated with both SortA-CD1d and SortA-TD-CD1d (**Figure 7**), with no difference in lipid abundance between SortA-CD1d and SortA-TD-CD1d. Similar observations were made for other, less abundant CD1d-associated lipids (**Figure 7**). As such, under constitutive conditions, and in the specific cell line studied, endolysosomal trafficking does not have a major effect on the spectrum and abundance of lipids associated with CD1d.

## Discussion

NKT cells are potent immune cells and their activation by CD1d-restricted antigen presentation therefore requires tight control (1–5). The balance between activating lipids (lipid antigens) and non-activating lipids bound to CD1d contributes to the regulation of NKT cells and previous work has identified CD1d-restricted lipid antigens as well as regulatory (non-activating) lipids associated with CD1d (1, 5, 21–23, 49–58). However, comprehensive, unbiased analysis of the spectrum of lipids associated with CD1d has remained challenging due to technical hurdles associated with the purification of CD1d. As such, while the analysis of peptides bound to MHC class I and II is typically achieved through detergent-based extraction of these proteins (59–61), similar procedures are not feasible for the study of CD1d-associated lipids as the use of detergents will release bound lipids. The study of CD1d-associated lipids has therefore largely relied on *in vitro* systems based on overexpression of truncated CD1d proteins released into the cell culture supernatant after passage through the secretory pathway (20, 21, 24, 53). While these approaches have provided important insight into the repertoire of lipids associated with engineered, secreted forms of CD1d, truncated CD1d does not undergo endolysosomal trafficking and can therefore not be used to study lipids loaded in these compartments. Moreover, secCD1d does not allow to study lipids associated with CD1d *in vivo* or *ex vivo*. Approaches for protease-cleavable CD1d proteins were developed (22, 23), but again faced technical challenges as these engineered proteins were either not cleaved as intended (23) or underwent spontaneous release in the absence of protease exposure (22). Here, we have therefore developed a SortA-based system for simultaneous harvesting and affinity tagging of CD1d proteins from mammalian cells. SortA-CD1d retains the original domain structure and subcellular trafficking patterns of endogenous CD1d, is able to load lipids in the endolysosomal system and can present these lipids to NKT cells. Of note, this system may also provide the opportunity to study CD1d-associated lipids *in vivo* or *ex vivo* through integration of the SortA recognition motif into the endogenous CD1d locus in animal models.

Using a mouse cell culture system expressing SortA-CD1d and SortA-TD-CD1d, we made several important and unexpected findings. First, while previous work had suggested hundreds of lipids to be associated with CD1d (21, 24), we showed, through the use of conventional MHC class I as control and stringent filtering criteria, that CD1d is predominantly associated with a limited number of long-chain sphingolipids and glycerophospholipids. These lipids are almost exclusively comprised of non-NKT cell-activating lipids including major negative regulators of NKT cell activation such as sphingomyelin (51). These findings are in line with the concept of tight control of NKT cell activation under constitutive conditions and suggest that sphingomyelin in particular acts as a central, physiologic inhibitor of NKT cell activation (51). Second, the spectrum and abundance of lipids associated with CD1d is, in contrast to previous suggestions (22, 23), not reminiscent of the total cellular lipidome (**Figures 6**, **7**). Instead, we observed no correlation between the abundance of lipids in the cellular lipidome compared to those associated with CD1d. As such, CD1d-associated lipids primarily comprised sphingo- and glycerophospholipids which are of modest to low abundance in the total cellular lipidome. Preferential association of CD1d with long-chain lipids may result from the fact that longer hydrophobic carbon chains provide increased stability of CD1d-lipid complexes (48). In addition, it is possible that lipid transfer and editing proteins involved in lipid processing and loading onto CD1d actively shape the spectrum of lipids associated with CD1d and thus contribute to a bias towards long-chain lipids. We cannot exclude that predominant association of CD1d with long-chain lipids at least partially results from the extraction and purification procedure, which may select for long-chain lipids due to their lower off-rates (48). This does not explain, however, the preferential association of CD1d with SM and Cer instead of more abundant cellular lipids such as PC, which is unlikely to be a consequence of the extraction procedure. Furthermore, while it is conceivable that the application of filtering criteria may have eliminated lipids truly associated with CD1d, filtering is unlikely to introduce a systemic bias towards long-chain lipids. Moreover, the application of less stringent filtering criteria neither affected the number nor classes of lipids associated with CD1d (**Figures 7A, B**). Besides carbon chain length, binding of lipids to CD1d is further dependent on saturation in the acyl chain structure (48, 62). McCarthy et al. proposed that a kink in the acyl chain of C20:2 due to unsaturated bonds stabilizes lipid binding in the curved A´ channel of CD1d, thus explaining the reduced dissociation rate of C20:2 in comparison to C20 (α-GalCer analogue with 20 carbon saturated N-acyl chain) (48). It is therefore possible that similar mechanisms contribute to increased affinity of SM 42:2:2 compared to other long-chain SMs, such as SM 42:1:2. A similar hypothesis for the preferred binding of SM 42:2 in comparison to 42:1 to CD1a was recently presented by Cotton et al. (63).

The majority of previous studies that assessed CD1d-associated lipids used truncated, secreted CD1d proteins, which do not undergo endolysosomal trafficking. Therefore, little is known about the contribution of endolysosomal CD1d trafficking to the spectrum and abundance of CD1d-associated lipids. Two reports investigated lipids (23) and glycosphingolipids (22) associated with full-length, cleavable CD1d molecules. Yuan et al. reported that a full-length CD1d constructs (pclCD1d) predominantly bound major cellular lipids such as PC 34:1, while secCD1d predominantly associated with SMs. The authors therefore suggested that CD1d loads SMs in the secretory pathway, which are subsequently replaced by glycerophospholipids in the endolysosomal pathway. However, extraction and purification procedures differed between pclCD1d and secCD1d, which may have affected lipidomics results. Muindi et al. compared secreted and TEV protease-cleavable CD1d proteins and analyzed CD1d-associated GSLs by HPLC. GSLs associated with TEV-CD1d more closely resembled the cellular GSL composition compared to secCD1d, but again, purification procedures differed for the two constructs. Our SortA-based approach allowed to comparatively assess the spectrum of lipids associated with SortA-CD1d and SortA-TD-CD1d, two constructs which differ in their ability to undergo endolysosomal trafficking, but were extracted and purified in an identical manner. Intriguingly, this work did not reveal differences in the lipidome associated with CD1d compared to TD-CD1d, suggesting that at least in this cell culture system under constitutive conditions, contributions of endolysosomal trafficking to the shaping of the CD1d-associated lipidome are limited. It is important to note that these observations were made under cell culture conditions in the absence of major sources of structurally distinct exogenous lipids (e.g. microbiota-derived lipids). As such, our findings do not rule out important contributions of the endolysosomal pathway to the loading of exogenous lipids *in vivo*. Moreover, while secCD1d proteins are secreted after passage through the secretory pathway and are thus fully excluded from the endolysosomal system, a minor fraction of TD-CD1d shows residual co-localization with these compartments (**Figure 2**). As such, it is possible that residual trafficking of TD-CD1d to endolysosomal compartments diminishes potential differences in the spectrum and abundance of lipids associated with CD1d compared to TD-CD1d.

Of note, our study has investigated CD1d-associated lipids solely in a single cell line under constitutive conditions *in vitro*. We anticipate that the spectrum of CD1d-associated lipids is affected by the cellular repertoire of lipids and possibly other cell type-specific effects such as differences in endolysosomal trafficking, lipid transfer activity, and accessibility of lipids in different subcellular compartments. As such, we cannot exclude, and in fact consider it likely, that the spectrum and abundance of CD1d-associated lipids will be different if analyzed in a different cell type. In addition, we anticipate that CD1d-associated lipidomes will show temporal changes in response to environmental exposures (e.g. diet, infection) and possibly other processes such as aging. The tools generated in our work will allow to address these questions in the future through the expression of cleavable CD1d in different cell types maintained under different conditions or through expression of cleavable CD1d in animal models *in vivo*.

One potential limitation of our work is that the molar amount of CD1d in each purification exceeded the total molar amount of CD1d-associated lipids in these fractions (**Figures 7A, B**). Several factors likely contributed to these observations. First, in affinity purification, molecules associated with purified proteins will invariably be partially lost during purification steps. As such, it is not anticipated that the sum of molar amounts of lipids identified will equal that of input protein. Second, it is possible that ELISA-based approaches, despite the use of appropriate standards, may not fully accurately determine CD1d quantity. In line with these considerations, quantitative proteomics of CD1d in similar purifications indicated lower amounts of CD1d compared to ELISA (**Supplementary Table 2**) corresponding to higher molar fractions of associated lipids. Third, the application of pre-specified filtering criteria may have excluded lipids truly associated with CD1d. However, while total molar amounts of lipids in the absence of filtering indeed added up to 80-90% of the molar amount of CD1d in these fractions (**Supplementary Table 1**), the vast majority of these lipids were (i) of very low abundance, (ii) not reproducibly associated with CD1d across different runs, and (iii) of similar abundance in H-2Kb purifications. This suggests that many of these lipids indeed represented contaminations rather than lipids truly associated with CD1d, which further supports the value of including MHC class I controls and applying stringent filtering criteria. However, we cannot exclude that our analysis missed potentially less abundant but functionally relevant CD1d-associated lipids. This includes complex glycosphingolipids, which were not the focus of our analysis.

In conclusion, we have developed a SortA-based system for the extraction and purification of intact CD1d proteins from live mammalian cells for the analysis of CD1d-associated lipids. Using this system, we demonstrate that CD1d preferentially associates with non-NKT cell-activating, long-chain sphingolipids and glycerophospholipids which predominantly load in the secretory pathway. Further work is required to investigate whether introduction of the SortA recognition motif into the endogenous CD1d locus of animal models enables the study of lipids bound to CD1d *in vivo*.

## Data Availability Statement

The data presented in the study are deposited in the ProteomeXchange repository, accession numbers are PXD034366 and PXD034495 (https://panoramaweb.org/CD1d.url).

## Author Contributions

MR, YW, TS, and EH-C, generated cell lines. MR and TS performed IF experiments and antigen presentation assays. GB generated lipids. MR and YW performed flow cytometry, sortagging and protein purification experiments. YW extracted lipids and performed shotgun lipidomics. MR and YW designed and planned experiments. LD and GG provided expression constructs for SortA and advise on engineering of SortA-cleavable CD1d. MR, YW, and SZ analyzed results, composed all figures and interpreted data. AndS and SZ coordinated and supervised the study. MR, YW, and SZ wrote the manuscript with input from all coauthors. AnnS performed proteomics experiments. All authors (MR, YW, TS, EH-C, LD, GG, GB, AnnS, AndS, and SZ) reviewed the manuscript and contributed to edits. All authors contributed to the article and approved the submitted version.

## Funding

This work was supported by the European Research Council (ERC Starting Grant agreement no. 336528), the Deutsche Forschungsgemeinschaft (DFG) (ZE 814/4-1, ZE 814/7-1), the DFG Excellence Cluster Center for Regenerative Therapies Dresden, and the Else Kröner Fresenius Stiftung (2015_A62) (to SZ).

## Conflict of Interest

Author GG was employed by Maze Therapeutics.

The remaining authors declare that the research was conducted in the absence of any commercial or financial relationships that could be construed as a potential conflict of interest.

## Publisher’s Note

All claims expressed in this article are solely those of the authors and do not necessarily represent those of their affiliated organizations, or those of the publisher, the editors and the reviewers. Any product that may be evaluated in this article, or claim that may be made by its manufacturer, is not guaranteed or endorsed by the publisher.
