# Supplementary material for: Sortase A-Cleavable CD1d Identifies Sphingomyelins as Major Class of CD1d-Associated Lipids
Source: Front Immunol. 2022 Jul 7;13:897873. doi: 10.3389/fimmu.2022.897873 (PMC9301999; doi:10.3389/fimmu.2022.897873)
Supplement: Supplementary file 1 [file DataSheet_1.docx]

Supplementary Material

# MS-based quantification with labelled peptide standards

Proteins in the aqueous phase of MTBE extraction were reduced with dithiothreitol, alkylated with iodacetamid and digested with trypsin (modified, sequencing grade, Promega). Resulting peptide mixtures were spiked with labelled peptide standards (New England Peptides) with final concentration 10fmol/μl and analyzed by LC-MS/MS. The analysis was performed on a nano-UPLC Ultimate 3000 interfaced on-line to a Q Exactive HF Hybrid Quadrupole Orbitrap mass spectrometer (both Thermo Fischer Scientific). The UPLC system was equipped with Acclam PepMap^tm^ 100 75 µm x 2cm trapping column and 75µm x 50cm separating column packed with 3 µm diameter C18 particles (Thermo Fischer Scientific). Peptides were separated using 80min linear gradient (solvent A – 0.1% formic acid in water, solvent B – 0.1% formic acid in acetonitrile). Spectra were acquired in parallel reaction monitoring (PRM) mode, a full mass spectrum at 240000 resolution (AGC target 3e6, 150 ms maximum injection time, m/z 350–1700) was followed by PRM scans at 120000 resolution (AGC target 1e5, 200 ms maximum injection time) triggered by a scheduled inclusion list. To set up PRM parameters, the information about target peptides (m/z, charge state, retention time) was obtained from prior experiments with recombinant proteins. Stability of labelled peptide standards was tested in a separate LC-MS/MS experiment. Data processing was performed by SkyLine software (1) , quantitative values were calculated based on sum of extracted peak areas of precursor and two isotopes.

1. MacLean B, Tomazela DM, Shulman N, Chambers M, Finney GL, Frewen B, et al. Skyline: an open source document editor for creating and analyzing targeted proteomics experiments. Bioinformatics. 2010 Apr 1;26(7):966–8.
